# Supplementary material for: Umbrella review of meta-analyses on the risk factors, protective factors, consequences and interventions of cyberbullying victimization
Source: Nat Hum Behav. 2024 Nov 8;9(1):101–32. doi: 10.1038/s41562-024-02011-6 (PMC11774762; doi:10.1038/s41562-024-02011-6)
Supplement: Supplementary file 1 — Reporting Summary [file 41562_2024_2011_MOESM1_ESM.pdf]

nature portfolio

Corresponding author(s): DAPRNATHUMBEHAV-23103327A

Last updated by author(s): Aug 27, 2024

Reporting Summary

Nature Portfolio wishes to improve the reproducibility of the work that we publish. This form provides structure for consistency and transparency in reporting. For further information on Nature Portfolio policies, see our Editorial Policies and the Editorial Policy Checklist.

Statistics

For all statistical analyses, confirm that the following items are present in the figure legend, table legend, main text, or Methods section.

n/a	Confirmed	
☒	☐	The exact sample size (*n*) for each experimental group/condition, given as a discrete number and unit of measurement
☒	☐	A statement on whether measurements were taken from distinct samples or whether the same sample was measured repeatedly
☒	☐	The statistical test(s) used AND whether they are one- or two-sided<br>*Only common tests should be described solely by name; describe more complex techniques in the Methods section.*
☒	☐	A description of all covariates tested
☒	☐	A description of any assumptions or corrections, such as tests of normality and adjustment for multiple comparisons
☐	☒	A full description of the statistical parameters including central tendency (e.g. means) or other basic estimates (e.g. regression coefficient) AND variation (e.g. standard deviation) or associated estimates of uncertainty (e.g. confidence intervals)
☒	☐	For null hypothesis testing, the test statistic (e.g. *F*, *t*, *r*) with confidence intervals, effect sizes, degrees of freedom and *P* value noted<br>*Give P values as exact values whenever suitable.*
☒	☐	For Bayesian analysis, information on the choice of priors and Markov chain Monte Carlo settings
☒	☐	For hierarchical and complex designs, identification of the appropriate level for tests and full reporting of outcomes
☐	☒	Estimates of effect sizes (e.g. Cohen's *d*, Pearson's *r*), indicating how they were calculated

Our web collection on statistics for biologists contains articles on many of the points above.

Software and code

Policy information about availability of computer code

Data collection	No software was used for data collection.
Data analysis	In the literature search for the systematic review, Mendeley Desktop version 1.19.8 (Mendeley, n.d.) was used in order to remove duplicates from the records obtained after the retrieval process. In cases where effect sizes were not reported in the form of correlations, conversions were conducted using R version 3.6.3 (R Core Team, 2020). effectsize version 0.0.6.1 (Ben-Shachar et al., 2020) was used to convert Cohen's d and odds ratios to Pearson's r. psych version 2.2.5 (Revelle, 2021) was used to convert Fisher's z to Pearson's r. In cases where Hedge's g was provided, it was converted to Cohen's d using the following formula, d= g/((1- 3/(4(n_1+ n_2)-9))) (http://dlinares.org/cohend.html), where n1 and n2 refer to the sample sizes of the two groups used to calculate the effect size. The result was then converted into Pearson's r using the effectsize package.

For manuscripts utilizing custom algorithms or software that are central to the research but not yet described in published literature, software must be made available to editors and reviewers. We strongly encourage code deposition in a community repository (e.g. GitHub). See the Nature Portfolio guidelines for submitting code & software for further information.

Data

Policy information about availability of data

All manuscripts must include a data availability statement. This statement should provide the following information, where applicable:

- Accession codes, unique identifiers, or web links for publicly available datasets
- A description of any restrictions on data availability
- For clinical datasets or third party data, please ensure that the statement adheres to our policy

The R analytic code used to convert effect sizes as well as all screening records of the current review are publicly available on Researchbox #1364 (https://researchbox.org/1364&PEER_REVIEW_passcode=FWAIHM).

Research involving human participants, their data, or biological material

Policy information about studies with human participants or human data. See also policy information about sex, gender (identity/presentation), and sexual orientation and race, ethnicity and racism.

Reporting on sex and gender	The analysis was conducted from data extracted from the meta-analyses included in the umbrella review. All references to sex and gender were in-line with how each meta-analyses referred to the variables.<br><br>The effect of gender as a predictor of cyberbullying victimisation was narratively synthesised within the results.
Reporting on race, ethnicity, or other socially relevant groupings	The analysis was conducted from data extracted from the meta-analyses included in the umbrella review. All references to age, race and ethnicity were in-line with how each meta-analyses referred to the variables.<br><br>The effect of age and race as predictors of cyberbullying victimisation was narratively synthesised within the results.
Population characteristics	No data was collected for the study as the research utilised a systematic review design. Data were extracted from the 56 meta-analyses included in the systematic review. Sample sizes of the included meta-analyses ranged from 421 to 1,136,080 (Mdn=53,183), covering all regions including Africa, Arab States, Asia-Pacific, Europe, Middle East, North America and South America. 50 records (89%) were journal articles, while 1 record (2%) was a book chapter, 1 record (2%) was a conference piece, and 4 records (7%) were dissertations/theses. Out of the 56 included records, 47 records (84%) focused specifically on children and/or adolescents and young adults (including those focused on school settings), and 6 records (11%) focused on both children/adolescent and adult samples, while only 1 record (2%) focused solely on an adult sample (2 records did not provide information on their participant type). Statistics regarding sample age or female proportion were not provided by the majority of the meta-analyses.
Recruitment	No participants were recruited as the research utilised an umbrella review methodology.
Ethics oversight	Ethical approval was not required as the study design (umbrella review) was exempted from the local Institutional Review Board.

Note that full information on the approval of the study protocol must also be provided in the manuscript.

Field-specific reporting

Please select the one below that is the best fit for your research. If you are not sure, read the appropriate sections before making your selection.

☐ Life sciences ☒ Behavioural & social sciences ☐ Ecological, evolutionary & environmental sciences

For a reference copy of the document with all sections, see nature.com/documents/nr-reporting-summary-flat.pdf

Behavioural & social sciences study design

All studies must disclose on these points even when the disclosure is negative.

Study description	The study utilizes an umbrella review method, and conducts an umbrella review of meta-analyses on the predictors and consequences of cyberbullying victimisation.
Research sample	A total of 56 records were included in the final review. Records were made available from 2012 to 2024 inclusive, and included meta-analyses covering studies from 1993 to 2023 inclusive. Sample sizes ranged from 421 to 1,136,080 (Mdn=53,183), covering all regions including Africa, Arab States, Asia-Pacific, Europe, Middle East, North America and South America. 50 records (89%) were journal articles, while 1 record (2%) was a book chapter, 1 record (2%) was a conference piece, and 4 records (7%) were dissertations/theses.
Sampling strategy	A search strategy was developed by the first author and agreed upon by the first, second and last authors in order to capture relevant records from each of the sources. Systematic searches were conducted by the first author on various sources for meta-analyses available up to 7 April 2024. Main sources comprised five databases (EBSCOhost ERIC, EBSCOhost PsycInfo, PubMed,

Scopus, Web of Science) and 13 journals related to the field of cyberbullying (Adolescent Research Review; Aggression and Violent Behavior; Aggressive Behavior; Children and Youth Services Review; Computers in Human Behavior; Cyberpsychology, Behavior, and Social Networking; Deviant Behavior; Journal of Adolescence; Journal of Pediatric Nursing; Journal of School Violence; New Media and Society; School Psychology Review; Trauma, Violence, & Abuse). The journals were selected based on search strategies of previous meta-analyses on the topic (Kowalski et al., 2014)) as well as by selecting journals that had recently published meta-analyses on the field of cyberbullying. To supplement the research, two other sources (ProQuest Dissertations and Theses, Google Scholar) were also searched to retrieve additional published literature as well as relevant unpublished literature.

The following keywords were used to conduct the systematic literature search within the five databases: ("meta-analy*" OR "meta analy*" OR "quantitative synthesis" OR "review*") AND (cyber* OR internet OR net OR online OR chat OR electronic OR mobile OR "social network" OR media OR Facebook OR Twitter OR Blog* OR Youtube OR Tumblr OR Discord OR Reddit OR Instagram OR Tiktok OR Snapchat OR Pinterest OR LinkedIn) AND (harass* OR bully* OR bulli* OR victim* OR aggres* OR abus* OR maltreat* OR incivil* OR toxic* OR violen* OR delinquen* OR devian* OR ragging OR hazing OR mobbing OR intimidat*). A simplified search string containing the following keywords was used to search the relevant journals and other sources: (meta-analysis OR "meta analysis" OR review) AND (cyber OR internet OR online OR "social media") AND (bully OR victim).

Data collection

The initial search returned 1583 records, of which 1149 remained after the removal of duplicates. Title and abstract screening resulted in the removal of a further 818 records. Full text-screening resulted in the removal of 331 records, leaving a final total of 56 records.

The following information was independently extracted from the final list of included meta-analyses by either the first and third author or the first author and a research assistant: author(s), year of publication, title of publication, countries and regions covered by the review, participant demographics, total number of studies, total unique sample size, cyberbullying definition and type of cyberbullying victimisation measured, predictors and/or consequences of cyberbullying victimisation, and the relevant effect sizes denoting the association between cyberbullying victimisation and the predictor and/or consequence of cyberbullying victimisation explored within each meta-analysis. Regional classification of the different countries followed the listing by Wikimedia, Meta-Wiki (2022). Effect sizes were extracted as given within each meta-analysis without any conversions.

Timing

The literature search was conducted for all papers published up to 7th April 2024.

Data exclusions

No data were exlucded

Non-participation

No participants dropped out.

Randomization

Participants were not allocated to experimental groups.

Reporting for specific materials, systems and methods

We require information from authors about some types of materials, experimental systems and methods used in many studies. Here, indicate whether each material, system or method listed is relevant to your study. If you are not sure if a list item applies to your research, read the appropriate section before selecting a response.

Materials & experimental systems

n/a	Involved in the study
☒	☐ Antibodies
☒	☐ Eukaryotic cell lines
☒	☐ Palaeontology and archaeology
☒	☐ Animals and other organisms
☒	☐ Clinical data
☒	☐ Dual use research of concern
☒	☐ Plants

Methods

n/a	Involved in the study
☒	☐ ChIP-seq
☒	☐ Flow cytometry
☒	☐ MRI-based neuroimaging

Plants

Seed stocks

Report on the source of all seed stocks or other plant material used. If applicable, state the seed stock centre and catalogue number. If plant specimens were collected from the field, describe the collection location, date and sampling procedures.

Novel plant genotypes

Describe the methods by which all novel plant genotypes were produced. This includes those generated by transgenic approaches, gene editing, chemical/radiation-based mutagenesis and hybridization. For transgenic lines, describe the transformation method, the number of independent lines analyzed and the generation upon which experiments were performed. For gene-edited lines, describe the editor used, the endogenous sequence targeted for editing, the targeting guide RNA sequence (if applicable) and how the editor was applied.

Authentication

Describe any authentication procedures for each seed stock used or novel genotype generated. Describe any experiments used to assess the effect of a mutation and, where applicable, how potential secondary effects (e.g. second site T-DNA insertions, mosiacism, off-target gene editing) were examined.
